# Prevalence and associated factors of prelacteal feeding among neonates admitted to neonatal intensive care units, North central Ethiopia, 2019

**DOI:** 10.1186/s12889-020-09578-5

**Published:** 2020-09-25

**Authors:** Wubet Alebachew Bayih, Demewoz Kefale Mekonen, Solomon Demis Kebede

**Affiliations:** Department of Pediatrics and Neonatal Health Nursing, College of Health Sciences, Debre Tabor University, P.O.BOX 272 Debre Tabor, Ethiopia

**Keywords:** Prevalence, Factors, Prelacteal, Neonates, Ethiopia

## Abstract

**Background:**

Prelacteal feeding compromises the benefits of exclusive breastfeeding, thereby increasing neonatal malnutrition, infection and mortality. About 26% of the Ethiopian neonates are given prelacteal feeds and nearly 48% of whom are attributed to Amhara region. Besides, prior Ethiopian studies have shown significance of the problem at different community settings in the country. However, the prevalence and associated factors of prelacteal feeding among neonatal admissions has been overlooked in the country in general and in the study area in particular. Therefore, this study was aimed to assess the prevalence and associated factors of prelacteal feeding among neonatal admissions in the study setting.

**Methods:**

A cross-sectional study was conducted among 321 mother-neonate pairs admitted to Neonatal Intensive Care Unit (NICU) of Debre Tabor General Hospital between September 2018 and February 2019. Every fourth mother neonate pair was included to the study. Multivariable logistic regressions were fitted to find out adjusted odds ratios (AORs) of factors associated with pre-lacteal feeding.

**Results:**

The prevalence of prelacteal feeding was 20.2% [95% CI: 15.31–26.75%]. Plain water was the most common prelacteal food (32.3%). Factors associated with prelacteal feeding include rural residence (AOR = 4.07, 95% CI: 1.30, 12.81), primiparity (AOR = 4.50, 95% CI: 1.30–12.81), less than four ANC visits (AOR = 4.71, 95% CI: 1.23–17.84), spousal accompany to ANC (AOR = 0.20, 95% CI: 0.05–0.75), home delivery (AOR = 5.94, 95% CI: 1.80–19.67), having twin newborns (AOR = 6.69, 95% CI: 1.25, 35.91) and maternal belief on the purported advantages of prelacteal feeding (AOR = 2.74, 95% CI: 2.09–25.39).

**Conclusion and recommendation:**

One out of five sick neonates was given prelacteal feeds. Twin neonates, home delivered neonates, rural neonates, neonates born to primiparous mothers, neonates delivered from mothers who had less than four ANC visits, neonates born to mothers who weren’t accompanied by their spouse during ANC and those born to mothers who believe on the purported advantages of prelacteal feeding had higher odds of receiving prelacteal feeds. Therefore, mothers of these vulnerable neonates should be provided with more emphasis about counseling of infant and young child feeding practice during their stay at NICU.

## Background

The World Health Organization and United Nations Children’s Fund recommend an exclusive and early initiation of breastfeeding following birth [1, 2]. This recommendation of optimal breastfeeding is however contravened by prelacteal feeding, which is a nutritional malpractice of providing newborns with any food before breast feeding during the first 3 days of birth. If a newborn is prelacteal fed, it isn’t exclusively breastfed and hence the nonexclusive breastfeeding in the first 3 days of neonatal life is often followed by giving further prelacteal foods resulting in suboptimal breastfeeding (i.e forming a vicious cycle of prelacteal feeding and suboptimal breastfeeding) [2–5].

As of the 2013 Ethiopian Health and Nutrition Research Institute appraisal of the achievements and identified gaps about status of infant and young child feeding (IYCF) practice policy and programs, the implementation of Baby friendly Hospital Initiatives (BFHI) was zero (0%). Therefore, since then, different programs like health extension programs and working in collaboration with non-governmental organizations have been extensively implemented [6]. Moreover, though there is no Ethiopian hospital currently certified as “Baby Friendly Hospital”, many hospitals in the country including our study hospital have been following the ‘Baby-Friendly Hospital Initiatives’. For example, the ten steps of successful breastfeeding have been given emphasis. However, there are still several myths towards exclusive breast feeding in Ethiopia thereby challenging the implementation of the ‘Baby-Friendly Hospital Initiatives’ in several areas of the country (including our study setting) [7]. More specifically, participants of this study were only sick neonates (both home and health facility delivered neonates) whose mothers might have given them prelacteal foods at home or on the way to NICU before admission. Thus, influence of the hospital setting was thought to be lower than it would be if the study had been among mother-neonate pairs in the postnatal ward. Prelacteal feeding is a major bottleneck to optimize neonatal survival because a prelacteal food is nutritionally inferior and deprives the neonate of the most valuable perfect combination of proteins, fats, carbohydrates, minerals and fluids in breast milk [1, 2]. Furthermore, if breastfeeding is replaced by prelacteal foods, the physiologic pattern of breast milk production becomes suppressed thereby compromising both the quality and quantity of anti-infective proteins that in turn increase neonatal susceptibility to different infections. Contamination of the foods and utensils used for introducing prelacteal foods can also increase neonatal vulnerability to infections especially due to permeability of their immature gut lining [3–6]. Therefore, prelacteal feeding increases the risk of early neonatal morbidity and mortality by limiting the health and emotional benefits of optimal breastfeeding [1, 3]. Annually, nearly 1.4 million under five deaths could be attributed to suboptimal breastfeeding [2]. Studies also showed that survivors of early nutritional deficits from prelacteal feeding are more likely to have poor intellectual performance, adverse reproductive outcomes [8] and chronic non-communicable diseases in their latter lives [9].

The practice of prelacteal feeding is common in Ethiopia contributing to 70,000 infant deaths per year, i.e., 24% of the total infant death annually [7]. Despite its harmful effects, prelacteal feeding is a deep rooted common nutritional malpractice that raises global concern. For example, country wise, its prevalence was: Jammu and Kashmir, India (88.0%), Vietnam (73.3%), Nepal (26.5%), western Uganda (31.3%), Nigeria (60.5%), Mansoura**,** Egypt (58%) and Ethiopia (26.95%) [10–16]. Moreover, the 2016 Ethiopian Demographic and Health Survey (EDHS) has shown that 25.9% of the neonates were given different prelacteal feeds. These feeds were: plain water (12.6%), non milk liquids like juice, juice drinks, clear broth or other liquids (2.6%), cow milk (1.5%) and solid or semi-solid complementary foods (3.1%). The demographic survey has also shown that Amhara regional state (a region of the study area) had the highest (47.8%) prevalence of pre-lacteal feeding practice next to Somali region (72.5%) in the country [17].

Community based studies conducted at different parts of Ethiopia have shown burden of prelacteal feeding along the spectrum of the national pediatric nutrition system; for instance, in Raya Kobo town (11.1%), Motta town (20.3%), Afar region (42.9%), Harar city (45.4%), Fiche town (24.4%), Aksum town (10.1%), Sidama zone (25.5%) and Debre Markos town (19.1%) [18–25]. Both the global and national studies have shown that common prelacteal foods include plain water [13, 22], raw butter [20], sugar (glucose) water, honey, tea and fruit juice [13, 16, 25]. The pre-lacteal use of these foods was found to be positively associated with many factors like delayed initiation of breast feeding [19–21, 25], home delivery [16, 21, 25], cesarean delivery [11, 23, 25], inability to read and write [12, 25], male neonate [25], maternal belief on the purported advantages of prelacteal feeding [23], less than four ANC visits [12, 15, 21, 23], previous experience of prelacteal feeding [25] and influence from somebody else [21]. The stated reasons to give pre-lacteal feeds were: to clean the infant’s stomach, to quench neonatal thirst, breast problems, maternal medical illness, inadequate breast milk secretion, colostrums is dirty, colostrums is inadequate for the neonate, being prelacteal fed is a guarantee of healthier growth in later life and presence of neonatal feeding problem [10–25]. An Ethiopian study at Raya Kobo district also showed that the main reason to give prelacteal feeds for sick neonates were to treat neonatal illness believing that their illness is attributable to ‘evil eye’ [18]. Furthermore, different studies in different Ethiopian towns namely at Ambo [26], Dabat [27], Mizan Aman [28] and Bedelle [29] revealed the presence of maternal knowledge and practice gap about WHO recommendations of exclusive breast feeding.

Despite the aforementioned community based evidence of prelacteal feeding in Ethiopia [18–25], there has been no prior study about the burden and associated factors of prelacteal feeding among neonatal admissions in the country in general and in the study area in particular. Therefore, in cognizance of the severity of prelacteal feeding among sick neonates, it is the authors’ firm conviction about significance of studying the prevalence and associated factors of prelacteal feeding practice by mothers of these neonates. Besides, public health importance of maternal residence, spousal accompaniment of ANC and having twin neonates are originally explored in this study. Findings of the study will be valuable to draw neonatal care providers’ attention towards maternal counseling of optimal Infant and Young Child Feeding (IYCF) practice during maternal stay at NICU. The counseling could help warrant safe IYCF after discharge thereby optimizing neonatal survival.

## Methods

### Study area and period

The study was conducted between September 2018 and February 2019 at Debre Tabor General Hospital, South Gondar Zone, Northcentral Ethiopia. The hospital is found 666 km far from Addis Ababa and 105 km away from Bahir Dar city. It is the largest hospital in South Gondar zone serving 2.7 million populations and linked to 7 district hospitals. Neonatal Intensive Care Unit (NICU) of the hospital had a total of 28 neonatal beds and hosted approximately 1159 admissions per year as per the averaged data from three subsequent quarters of 2018. Other than prematurity and perinatal asphyxia, many of the admissions were attributed to the complications of harmful traditional practices like traditional uvulectomy and suboptimal breastfeeding [30].

### Study design and participant characteristics

It was a hospital-based cross-sectional study. The study involved any sick neonate admitted to NICU (i.e both home delivered and those born at health facility were considered). NICU set up was purposively selected to show burden of prelacteal feeding among sick neonates of any birth place (home versus health facility) and any residence (urban versus rural). For this study, mothers were the source of data for most of the considered factors (both neonatal and maternal). Thus, maternal mental competency was the first and foremost criterion of inclusion. Besides, mothers’ ability to listen and speak was considered because interviews were conducted. Therefore, 3 mothers having post partal major depressive disorders, one mother with homicidal ideation, 7 mothers with eclamptic presentation and 5 mothers having natural impairment of talking/listening were excluded. Diagnosis of the aforementioned critical psychiatric illnesses was made by psychiatric professionals in the hospital.

### Sample size determination and sampling procedure

Sample size was determined using the following single population proportion formula.

$$ \mathbf{n}={\left(\mathrm{z}\ \left(\alpha /2\right)\right)}^2\mathrm{p}\ \left(1-\mathrm{p}\right)/{\mathrm{d}}^2. $$

Where**, n** = the minimum sample size required for the study,

**Z (α/2**) = the desired level of confidence interval which is 95% (1.96).

**P** = a reasonable estimate for the prevalence of prelacteal feeding from a study conducted in Southern Ethiopia (25.5%) [24].

**d** = tolerable margin of error, 5% (0.05).

Then, the calculated sample size became 292 and after adding 10% none response rate, the total sample size was 292 + 29 = 321.

Therefore, the total sample size considered for this study was 321 postnatal mothers. Using systematic sampling, every fourth (*K* = 1159/321 ≈ 4) eligible woman-neonate pair at NICU was selected systematically over 6 months to reach the most representative sample by systematically selecting as many diverse study subjects as possible.

### Data collection procedures

Data were collected by four well trained BSc neonatal nurses through face to face interview using a pretested questionnaire. An interview was conducted for eligible mothers at the time of admission to Neonatal Intensive Care Unit. The questionnaire contained factors related to maternal socio demography, neonatal health, Maternal and Child Health (MCH) service utilization and maternal information on prelacteal feeding. Once interviewed for the study, a mother was ensured not to be reconsidered by the time she comes at another time during the study period. This was done by putting a ‘√’ mark on the cover of the neonatal chart so that a mother-neonatal yard won’t be reselected if encountered again.

### Variables and measurement

#### Outcome variable

Prelacteal feeding: a neonate was regarded as prelacteal fed if given any anything before breastfeeding during the first 3 days of birth [17–25].

#### Explanatory variables

Utilized antenatal care: having at least one visit of health institution for checkup purpose during the pregnancy of the index neonate [1, 4].

Utilized postnatal care: having at least one visit of health institution for checkup of both maternal and neonatal health of the index neonate [1, 4].

Early initiation of breastfeeding: when the neonate was initiated to breast feed within 1 h of birth [1–5].

Late initiation of breastfeeding: when the newborn was initiated to breastfeed after 1 h of birth [1–5].

Premature: When a neonate was born below 37 completed weeks of intrauterine life [31].

Mature: when a neonate was born at [37–41^6/7^] weeks of intrauterine life [31].

Post date: When a neonate was born at ≥42 completed weeks of intrauterine life [31].

Primiparous: when a mother had only one live birth [32].

Multiparous: when a mother had more than one live birth [32].

Short: When the inter-birth interval between the immediate prior child and the index neonate is less than 3 years [33].

Optimal: When the inter-birth interval between the immediate prior child and the index neonate is ≥3 years [33].

Low birth weight refers to weight of the newborn less than 2500 g at birth [31].

### Data quality control methods

#### Data collection tool

The questionnaire was first prepared in English (Supporting information 1) and then translated to local language, Amharic (Supporting information 2), suitable for data collection. Two professional translators with medical experience were hired during data cleaning in order to explain the questions and correctly translate maternal answers for minimizing the risk of information loss during translation. The tool was adapted from Ethiopian and other African studies after which it was validated for reliability of measurement [1, 3, 15, 27].

One day training and clear orientation was first provided for data collectors and supervisors on the process of data collection. Before the actual data collection, the questionnaire was validated by pretesting on 16 eligible postnatal mothers (5% of sample size) at Nefas Mewucha District Hospital, which is nearby to the study hospital. After pretesting of the questionnaire, clarity of questions, wordings, sequence of questions, skip pattern and respondents’ reaction to the questions were reconsidered and modified.

During data collection, data collectors were closely monitored and guided by two MSc neonatal nurse supervisors for complete and appropriate collection of the data. Reporting of the collected data to the principal investigator was made on a daily basis. Furthermore, the collected data were double entered into Epidata version 4.2 by two data clerks for validation purpose.

### Statistical analysis

The collected data were coded, cleaned, edited and double entered into epidata version 4.2 after which it was exported to *stata* version 14 software for further transformation and analysis. Frequencies, proportion, summary statistics and cross tabulation were used to describe the study population in relation to relevant variables and findings were presented using text and tables. The assumptions for binary logistic regression model were first checked and then bivariable analysis was carried out to identify candidate variables for multivariable analysis at *P* < 0.25 [16, 18, 19, 21]. Then, multivariable logistic regressions were performed using the candidate variables to investigate factors which have either positive or negative odds of association [(*p* < 0.05 at 95% confidence interval (CI)] with prelacteal feeding after auto adjustment of confounding effect in the final model. From the final model, modes of delivery and initiation time of breastfeeding were found to have confounding effects. Multi-collinearity between the study variables was first diagnosed using standard error and correlation matrix. Besides, Hoshmer-Lemeshow statistic and Omnibus tests were performed, and Hosmer-Lemeshow’s test was found to be insignificant (*p*-value = 0.301) while Omnibus tests was significant (*P*-value = 0.000) indicating the model was fitted.

### Ethical consideration

Ethical clearance was obtained from ethical review committee of Debre Tabor University. Following the approval, official letter of co-operation was given to the hospital manager. After explanation of the study, an informed verbal voluntary consent was obtained from each postnatal mother. Moreover, the mothers were told that the information they gave was treated with complete confidentiality and do not cause any physical harm. Mothers were counseled of avoiding prelacteal feeding to ensure healthy growth of their neonates. All the interviewed mothers were counseled about the health, emotional and social advantages of exclusive breastfeeding of their neonates. Similarly, they were taught of the adverse consequences of prelacteal feeding like infection, malnutrition and decreased bonding of mothers to their neonates. The counseling was assisted by video show using local language (Amharic) to ease their understanding. Otherwise, there were no any other gifts provided to the mothers.

## Results

### Sociodemographic characteristics

A total of 321 postnatal mothers were participated in this study with a response rate of 100%. More than half of the mothers, 184 (57.3%) were rural residents. The mean maternal age was 26.2 years (SD = ± 4.3) with almost half of the mothers in the age group of 20–34 years. About three fourth of the mothers 246(76.6%) were married and nearly the same number of mothers 242(75.4%) were multiparous. Most of the mothers were orthodox Christian 262(81.6%) (Table 1).

**Table 1:** Socio-demographic characteristics of prelacteal feeding among postnatal mothers presenting to neonatal intensive care unit of Debre Tabor General Hospital, Amhara regional state, North Central Ethiopia, 2019 (*n* = 321)

Socio- demographic factors	Frequency	%
Residence		
Urban	137	42.7
Rural	184	57.3
Marital status		
Single	43	13.4
Married	246	76.6
Divorced	17	5.3
Widowed	15	4.7
Religion		
Orthodox Christian	262	81.6
Muslim	37	11.5
Protestant	22	6.9
Age (years)		
16–20	61	19.0
20–34	166	51.7
> 34	94	29.3
Level of education		
Unable to read and write	138	43.0
Read & write	80	24.9
Primary education (grades1–8)	61	19.0
Secondary education (grades 9–12)	23	7.2
Diploma and above	19	5.9
Average monthly income ($ USA)		
< 37.5	113	35.2
≥ 37.5	208	64.8
Parity		
Primiparous	79	24.6
Multiparaous	242	75.4
Birth spacing (*n* = 242)		
Short	98	40.5
Optimal	144	59.5

### Neonatal characteristics of prelacteal feeding

In this study, important neonatal characteristics like sex, maturity at birth, birth weight, postnatal age, whether the neonate was single/twin type and history of admission to NICU were considered. About one third of the neonates were premature 121 (31.9%) and nearly equal number of neonates had low birth weight 122(32.2%). Moreover, 116 (30.6%) neonates were twin type. The study also revealed the presence of 65 neonates (17.2%) with prior history of admission to neonatal intensive care unit (Table 2).

**Table 2:** Neonatal characteristics of the postnatal mothers presenting to neonatal intensive care unit at Debre Tabor General Hospital, Amhara regional state, North Central Ethiopia, 2019 (*n* = 379)

Factor	Frequency	%
Newborn type		
Single	263	69.4
Twin	116	30.6
Sex		
Male	175	46.2
Female	204	53.8
Postnatal age (days)		
< 7	163	43.0
≥ 7	216	57.0
Maturity at birth		
Premature	121	31.9
Mature	223	58.9
Postmature	35	9.2
Birth weight (grams)		
< 2500	122	32.2
≥ 2500	257	67.8
Admission history to NICU		
Yes	65	17.2
No	314	82.8

### Maternal and child health care service utilization

Majority of the mothers, 295(91.6%) had ANC follow up and about two third of them 194(65.8%) attended at least 4 times. However, more than a quarter of the overall postnatal mothers, 85(26.5%), delivered at home. Regarding postnatal service utilization, 269(83.8%) of the postnatal mothers received at least the first postnatal care (i.e. within 24 h of delivery). Moreover, out of 296 (92.2%) mothers who received counseling, only one third of them 97(32.8%) were counseled during their ANC follow up. Counseling was about colostrum feeding (90.7%), exclusive breastfeeding (88.8%), positioning and attachment (45.1%) and management of the likely breast complaints (41.4%) Nevertheless, there were 25(7.8%) mothers who didn’t get counseling of optimal breastfeeding during their antenatal and postnatal follow ups. Concerning the initiation time of breast feeding, 133(41.4%) postnatal mothers initiated lately (after an hour of birth) (Table 3).

**Table 3:** Healthcare service utilization-related factors of prelacteal feeding practice among postnatal mothers presenting at neonatal intensive care unit of Debre Tabor General Hospital, Amhara regional state, North Central Ethiopia, 2019 (*n* = 321)

Factors	Frequency	%
ANC visit (*n* = 321)		
Yes	295	91.9
No	26	8.1
Number of ANC Visits (*n* = 295)		
< 4	101	34.2
≥ 4	194	65.8
Spousal accompany to ANC (*n* = 246)		
Yes	86	35.0
No	160	65.0
Place of delivery		
Health institution	235	73.2
Home	86	26.8
Mode of delivery		
Vaginal	276	86.0
Cesarean section	45	14.0
Personnel who assisted delivery		
Health professional	236	73.5
Traditional birth attendant	85	26.5
Birth outcome		
Single	263	81.9
Twin	58	18.1
Initiation time of breast feeding		
Early	188	58.6
Late	133	41.4
Postnatal care (PNC) utilization		
Yes	269	83.8
No	52	16.2
Counseled on the principles of optimal breastfeeding		
Yes	296	92.2
No	25	7.8
If yes, when?(*n* = 296)^a^		
During ANC	97	32.8
During PNC	199	67.2
^a^what were you counseled about? (*n* = 296)		
Exclusive breast feeding up to 6 months	285	88.8
Colostrum feeding	291	90.7
Positioning and attachment	145	45.1
Management of breast complaints	133	41.4

^a^Multiple responses were given

PNC refers to mothers who received at least the first postnatal care (within 24 h of delivery)

### Prelacteal feeding practice of postnatal mothers

From the study, prelacteal feeding was practiced by 65 mothers (20.2%) [95% CI: 15.90–24.60]. Plain water (32.3%) was the most common prelacteal food in the study area. Even though majority of the mothers practiced prelacteal feeding by own decision, the influence of grandparents (33.9%) and traditional birth attendants (15.4%) was also reported (Table 4).

**Table 4:** Prelacteal feeding practice among postnatal mothers presenting to neonatal intensive care unit of Debre Tabor General Hospital, Amhara regional state, North Central Ethiopia, 2019

Factors	Frequency	%
Practiced Prelacteal feeding for the current neonate (*n* = 321)		
Yes	65	20.2
No	256	79.8
Type of the prelacteal food (*n* = 65)		
Plain water	21	32.3
Formula milk	19	29.3
Sugar water	11	16.9
Butter	8	12.3
Cow milk	6	9.2
Method of feeding (*n* = 65)		
Spoon	33	50.8
Finger	17	26.1
Stick	15	23.1
Who did influence you to give pre-lacteal feeds?(*n* = 65)		
Maternal own decision	27	41.5
Grandparents	22	33.9
Traditional birth attendants	10	15.4
Friends	6	9.2
History of prelacteal feeding (*n* = 321)		
Yes	37	11.5
No	284	88.5

### Maternal information of prelacteal feeding

About one third [100 (31.2%)] of the mothers were found to believe on the purported advantages of prelacteal feeding. Among these mothers, the majority [43(43.0%)] claimed that prelacteal feeds were given for their cultural value. Three fourth of the mothers [242(75.4%)] were able to mention the risks of prelacteal feeding of which diarrhoea 203(83.9%) was most mentioned. There were 28 (8.7%) mothers that didn’t provide their newborns with colostrum for various reasons. From these reasons, breast milk inadequacy was most common 13(46.4%) (Table 5).

**Table 5:** Information of prelacteal feeding among postnatal mothers presenting at neonatal intensive care unit of Debre Tabor General Hospital, Amhara regional state, North Central Ethiopia, 2019

Factors	Frequency	%
Believed on the purported advantages of prelacteal feeding (*n* = 321)		
Yes	100	31.2
No	221	68.8
Maternal reasons of their belief on the purported advantages of prelacteal feeding (*n* = 100)		
PLF has cultural value	43	43.0
PLF doesn’t cause neonatal thirst	23	23.0
PLF improves neonatal health and growth	15	15.0
PLF calms/soothes the baby	13	13.0
PLF cleans the newborn’s bowel, throat or mouth	6	6.0
Prelacteal feeding has risks (*n* = 321)		
Yes	242	75.4
No	79	24.6
^a^If yes, could you mention?(*n* = 242)		
Diarrhea	203	83.9
Poor growth	159	65.7
Infection	106	43.8
Vomiting	71	29.3
Colostrum feeding(*n* = 321)		
Yes	293	91.3
No	28	8.7
If no, why? (*n* = 28)		
Inadequate breast milk secretion	13	46.4
Maternal medical illness	8	28.6
Cause abdominal discomfort and diarrhea	7	25.0

^a^Multiple responses were given

### Factors associated with prelacteal feeding practice

From bivariable analysis, maternal residence, parity, number of antenatal care visits, spousal accompany to ANC follow up, place of delivery, mode of delivery, number of newborns per mother, initiation time of breastfeeding and maternal belief on the purported advantages of prelacteal feeding were significant factors. However, from multivariable analysis, seven of these factors namely residence, parity, number of ANC visits, spousal accompany to ANC follow up, place of delivery, the number of newborns per mother and maternal belief on the purported advantages of prelacteal feeding were found to significantly predict prelacteal feeding practice.

From adjusted regression analysis, the odds of prelacteal feeding among primiparous mothers were 4.5 times higher than for multiparous mothers (AOR = 4.50, 95% CI: 1.30–12.81). Moreover, the odds of practicing prelacteal feeding among mothers who had less than four ANC visits were 4.7 times higher as compared to those having four and above visits (AOR = 4.71, 95% CI: 1.23–17.84). However, spousal accompany to ANC follow up was found to reduce the likelihood of prelacteal feeding practice (AOR = 0.20, 95% CI: 0.05–0.75). The odds of prelacteal feeding among home delivered mothers were 5.9 times higher as compared to those who gave their birth at health facility (AOR = 5.94, 95% CI: 1.80–19.67). Regarding the number of live newborns per mother, the odds of giving prelaceal feeds among mothers with twin newborns were 6.7 times higher as compared to those owning single neonate (AOR = 6.69, 95% CI: 1.25, 35.91). The study also revealed that mothers who believed on the purported advantages of prelacteal feeding were 7.3 times higher to practice prelacteal feeding as compared to those who didn’t have the belief (AOR = 7.29, 95% CI: 2.09–25.39). Concerning residence, the odds of prelacteal feeding practice among rural mothers were 4.1 times higher than mothers of urban residence (AOR = 4.07, 95% CI: 1.30, 12.81) (Table 6).

**Table 6:** Factors associated with prelacteal feeding practice among postnatal mothers presenting to neonatal intensive care unit of Debre Tabor General Hospital, Amhara regional state, North Central Ethiopia, 2019

Factors	Prelacteal feeding		Odds Ratio at 95% CI		*P*-value
	Yes n(%)	No n(%)	COR	AOR	
Residence (*n* = 321)					
Urban	17 (26.2)	120 (47.0)	1.0	1.0	
Rural	48 (73.8)	136 (53.1)	2.50 (1.36, 4.56)	4.07 (1.30, 12.81)	.016
Parity(*n* = 321)					
Primiparous	49 (75.4)	30 (11.7)	23.07 (11.7, 45.6)	4.50 (1.53, 13.26)	.006
Multiparous	16 (24.6)	226 (88.3)	1.0	1.0	
ANC visits (*n* = 295)					
< 4	60 (92.3)	46 (20.0)	48.00 (18.24, 126.34)	4.71 (1.23, 17.84)	.022
≥ 4	5 (7.7)	184 (80.0)	1.0	1.0	
Spousal accompany to ANC (*n* = 246)					
Yes	10 (15.4)	76 (42.0)	0.25 (0.12, 0.52)	0.20 (0.05, 0.75)	.017
No	55 (84.6)	105 (58.0)	1.0	1.0	
Place of delivery(*n* = 321)					
Health facility	9 (13.8)	226 (88.3)	1.0	1.0	
Home	56 (86.2)	30 (11.7)	46.87 (21.06, 104.35)	5.94 (1.80,19.67)	.004
Mode of Delivery(*n* = 321)					
Vaginal	38 (58.5)	238 (93.0)	1.0	1.0	
Cesarean section	27 (41.5)	18 (7.0)	9.40 (1.57, 15.32)	1.40 (0.46, 4.23)	.553
Birth outcome (*n* = 379)					
Single	13 (20.0)	250 (97.7)	1.0	1.0	
Twin	52 (80.0)	64 (2.3)	15.63 (1.25, 18.69)	6.69 (1.25, 35.91)	.027
Initiation time of breastfeeding (*n* = 321)					
Early	9 (13.8)	179 (57)	1.0	1.0	
Late	56 (86.2)	77 (24)	14.47 (6.81, 30.71)	0.27 (0.05, 1.47)	.132
Belief on the purported advantages of prelacteal feeding (*n* = 321)					
Yes	56 (86.2)	44 (17.2)	30.68 (14.11, 66.68)	7.29 (2.09, 25.39)	.002
No	9 (13.8)	212 (82.8)	1.0	1.0	

## Discussion

The practice of giving prelacteal food to neonates is a traditionally accepted culture in the Ethiopian community despite its risks. In this study, mother-neonatal pairs visiting neonatal ICU were selected for the purpose of assessing the burden and associated factors of pre-lacteal feeding among sick neonatal admissions to the intensive care units. Assessing the prevalence and predictors of pre-lacteal feeding in these settings could provide neonatal care providers with focused counseling of the admitted mothers about optimal breastfeeding of their neonates to prevent further neonatal illness owing to prelacteal feeding after discharge. It was found that one out of five sick neonates (20.2%) was given prelacteal feeds. Moreover, rural residence, primiparity, less than four ANC visits, home delivery, giving twin birth and maternal belief on the purported advantages of prelacteal feeding were significant factors associated with increased odds of prelacteal feeding practice whereas spousal accompaniment of ANC reduced likelihood of the practice.

In this study, the prevalence of pre-lacteal feeding (20.2%) was lower as compared to Ethiopian studies at Afar (42.9%) [20] and Harari regions (45.4%) [21], and this variation might be due to differences in study period, setting and sample size. Moreover, the prevalence was lower than studies in India (88%) [10], Vietnam (73.3%) [11], Nigeria (60.5%) [14], western Uganda (31.3%) [13] and Egypt (58%) [15], and the variation may be attributed to sample size and cultural differences among the study populations of the respective studies. The prevalence of prelactal feeding in the current study also revealed about 5 % decline in less than 5 years compared to the EDHS report of 2016 (26.5%) [17]. The most likely reasons of such a decline could be due to hospital based setting of our study by employing a smaller sample size compared to the EDHS report that involved millions of Ethiopian neonates in the community setting. However, the prevalence of pre-lacteal feeding in the study area was higher than other Ethiopian studies at Aksum town (10.1%) [23] and Raya Kobo district (11.1%) [18], and it could be due to differences in study setting i.e. for this study, sick neonates admitted to NICU were considered. In this case, sick neonates might have received prelacteal feeds because it is obvious that they might not be able to be breastfed due to some complications.

Regarding prelacteal food type, plain water (32.3%) was predominantly given in the study area which was consistent with studies at Nepal [12], Vietnam [11] and Fiche (Ethiopia) [22] but different from results of other studies done in Nigeria [14], Raya Kobo [18], Aksum [23], Sidama [24] and Debre Markos towns [25] where the most commonly given prelacteal feeds were sugar solution, sugar or glucose water, boiled water, formula milk and cow milk. The possible reason for this discrepancy might be due to differences in study setting (i.e other Ethiopian studies used only urban residents while ours included both urban and rural neonatal population). Prelacteal feeds were given for several misconceived reasons. In this study, the main reason of mothers to practice prelacteal feeding was due to cultural habit. This was found congruent with an Egyptian study [15] and several Ethiopian studies [18–25]. The resemblance may be due to the deep rooted cultural correlation of prelacteal feeding practice with its misconceived advantages in the community.

Adjusting for other factors, the odds of prelacteal feeding among primiparous mothers were 4.5 times higher than for multiparous mothers and it was in line with a Nepalese study [12]. This may be due to the usual challenges faced by primiparous mothers regarding proper positioning and attachment. This is because when a mother fails to position and attach her neonate to the breasts properly, the neonate becomes malnourished and irritable. Just at this time, the mother feels that her neonate isn’t getting adequate breast milk and try to introduce prelacteal foods to quench its hunger feelings. This challenge is often attributed to the less knowledge and skill that primiparous mothers have about proper newborn feeding practice [2, 6, 7, 31–33]. They may also rely on the advice from older women in the household or community who often prefer the earliest introduction of traditional practices like prelacteal feeding [10, 15].

This study revealed that less number of ANC visits has a good opportunity for mothers to practice prelacteal feeding. Thus, the odds of prelacteal feeding among mothers who had less than four ANC visits were 4.7 times higher as compared to those having four and above visits. This finding was congruent with those studies conducted at Nepal [12], Egypt [15], Aksum [23] and Harar [21] where the odds of prelacteal feeding among mothers having less than 4 ANC visits were 2 to 10 times greater than those mothers having at least 4 ANC visits. The best likely reason for the congruency may be the less often a mother is exposed to ANC clinic, the less information on neonatal exclusive breast feeding she will get from ANC providers.

Regarding spousal accompaniment of ANC, mothers who were accompanied by their spouses to ANC were 80% less likely to practice prelacteal feeding as compared to those who came lonely. The possible justification may be due to the golden opportunity of accessing husbands to teach the couples about the harmful effects of prelacteal feeding. This taught body of knowledge can be talked about and shared among the couples in the sphere of early and exclusive breastfeeding of their neonates [31–33].

In principle, twin breastfeeding is similar with breastfeeding of singletons because breast milk supply works on the principle of supply and demand (i.e. with twin newborns, more milk is removed from the breasts, so more milk is produced). This demand - supply based breastfeeding helps ensure an exclusive breastfeeding [1, 2, 31–34]. However, in this study, the odds of prelacteal feeding among mothers who possessed twin newborns were 6.7 times higher as compared to those having single newborns. This may be due to the wrong belief that mothers have insufficient breast milk for two newborns, their inexperience in twin breastfeeding and inadequate knowledge of optimal breastfeeding. Moreover, it can be attributed to the sleepy nature and suckling problems often associated with low birth weight of twin newborns which in turn causes late initiation of breastfeeding. Therefore, during her ANC sessions, a twin pregnant mother should be counseled of ensuring good breast milk supply through proper positioning and attachment.

Home delivery was found to be associated with increased odds of prelacteal feeding as compared to mothers who gave their birth at health facility. The most practical explanation to support this current finding could be mothers who delivered at home are often influenced by traditional birth attendants to give prelacteal feeds. Moreover, most of these mothers may be deprived of the first postnatal visit so that they can’t get health professionals’ counseling and support of optimal breast feeding. This finding was consistent with prior Ethiopian studies at Harar city [21] and Debre Markos town [25] where the odds of prelacteal feeding among mothers who delivered at home were 3.4 times and 4.4 times higher than those who delivered at health institution respectively.

Concerning residence, being a rural mother was found to be a risk for prelacteal feeding practice because the odds of prelacteal feeding among rural mothers were found to be 4.1 times higher than urban mothers. This could be due to rural dwelling mothers had less access to different communication media that raises awareness about optimal breastfeeding and risks of prelacteal feeding. Moreover, rural mothers couldn’t get an easy access to various health institutions that integrate optimal breastfeeding in their ANC and PNC (post natal care) programs. Unlike this finding, the study reported from Egypt showed urban residence was a risk for prelacteal feeding practice [15].

Findings of this study are supposed to have substantial contribution for the promotion of optimal breastfeeding practices that serve as a spring board to achieve the sustainable development goal by reducing Ethiopian neonatal mortality rate to as low as 12/1000 live births by 2030. However, despite our data quality control measures, these findings could have been influenced due to interviewers’ bias, maternal socially desirable answers for some factors and maternal failure to recall of pertinent data like her age. Besides, despite very high home delivery in Ethiopia, the study considered only postnatal mothers presenting to NICU that might have introduced selection bias. Therefore, our estimate might not be close to the truth of the general population in the community. Moreover, the results may not be representative of the entire Ethiopian situation due to a smaller sample size. Being a cross sectional study, its ability to draw any causal inference was limited. Lack of support with qualitative data is also another limitation. Therefore, a community based follow up study with qualitative support is recommended to show wider picture of prelacteal feeding practice and to further understand its maternal reasons in the specific context of the study setting i.e. South Gondar Zone.

## Conclusions

Prelacteal feeding practice among sick neonates was found to be a problem of public health importance in the study area. Fortunately, its enablers were modifiable. Therefore, the existing programs of promoting optimal breastfeeding should be strengthened during routine peri-natal healthcare contacts of every mother. Moreover, mothers should be advocated of spousal accompaniment while implementing these programs to give couple counseling of early and exclusive breastfeeding. The existing compassionate and respectful caring approach should also be enhanced to increase number of ANC visits and institutional delivery rate.

According to different literatures, a mother’s breast milk is sufficient for multiple neonates. Therefore, mothers expecting twin birth should be given more due emphasis of optimal breastfeeding during their antenatal follow up. Lastly, the existing educational programs that involve community agents should be reinforced to bring behavioral change about misconceived advantages of prelacteal feeding practices.

## Supplementary information

**Additional file 1: Supporting information 1.** Survey questionnaire in English language.

**Additional file 2: Supporting information 2.** Survey questionnaire in Amharic (original) language.

## Data Availability

Data will be available upon request from the corresponding author.

## Abbreviations

ANC: Stands for Ante Natal Care whereas AOR for Adjusted Odds Ratio
BFHI: For Baby Friendly Hospital Initiative
COR: For Crude Odds Ratio
DTGH: For Debre Tabor General Hospital
EDHS: For Ethiopian Demographic health survey
SPSS: For Statistical Package for Social Sciences and WHO for World Health Organization
