# Supplementary material for: Prevalence and associated factors of prelacteal feeding among neonates admitted to neonatal intensive care units, North central Ethiopia, 2019
Source: BMC Public Health. 2020 Sep 25;20:1457. doi: 10.1186/s12889-020-09578-5 (PMC7519479; doi:10.1186/s12889-020-09578-5)
Supplement: Supplementary file 1 — Additional file 1: Supporting information 1. Survey questionnaire in English language. [file 12889_2020_9578_MOESM1_ESM.docx]

**English Version of the Questionnaire**

**Code No: _______**

| **Part I: Maternal socio-demographic characteristics** | | | | |
| --- | --- | --- | --- | --- |
| **No.** | | **Questions** | **Response** | |
| 1 | | Residence | 1. Urban 2. Rural | |
| 2 | | Marital status | 1. Single 2. Married 3. Divorced 4. Widowed | |
| 3 | | Rreligion | 1. Orthodox 2. Muslim 3. Protestant 4. Adventist 5. Others (specify)___ | |
| 4 | | Age | ___________ (years) | |
| 5. | | Maternal level of education | 1. Unable to read and write 2. Read & write 3. Primary (1-8) education completed 4. Secondary (9-12) education completed 5. Diploma & above | |
| 6. | | Average family monthly income | _____________ ($USA) | |
| 7. | | Parity | 1. Primiparous 2. Multiparous | |
| 8. | | Birth spacing | ___________ | |
| Part II: Neonatal profile of postnatal mothers | | | | |
| 9 | | Newborn type | Single  1. Twin 2. Triplet 3. Quadruplet | |
| 10 | | Sex | 1. Male 2. Female | |
| 11 | | Postnatal age | _______(days) | |
| 12 | | Maturity at birth | _______(weeks) | |
| 13 | | Birth weight | _______(grams) | |
| 14 | | Admission history | 1. Yes 2. No | |
| Part III: Factors related to maternal and child health care service utilization | | | | |
| 15 | Did you attend ANC clinic during your last pregnancy? | | | 1. Yes 2. No |
| 16 | If your answer to **Question 16** is yes, how many visits did you attend? | | | 1. One 2. Two 3. Three 4. Four 5. Greater than four |
| 17 | Were you accompanied by your spouse during ANC? | | | 1. Yes 2. No |
| 18 | Where did you give birth? | | | 1. Health institution 2. Home |
| 19 | What is your mode of delivery? | | | 1. Vaginal 2. Cesarean Section |
| 20 | Who did assist your birth? | | | 1. Health professionals 2. Traditional birth attendant 3. Others (specify)------------- |
| 21 | What was your birth outcome? | | | 1. Single 2. Twin 3. Triplet 4. Quadruplet |
| 22 | When did you initiate breastfeeding? | | | ________(hour) |
| 23 | Have you attended postnatal care? | | | 1. Yes 2. No |
| 24 | Were you counseled about the principles of breastfeeding? | | | 1. Yes 2. No |
| If yes to **Question 24**, answer the next two questions. | | | | |
| 25 | When were you counseled about the principles of breastfeeding? | | | 1. During antenatal care 2. During postnatal care |
| 26 | What were you counseled about? | | | 1. Initiation of breast feeding within an hour of birth 2. Exclusive breastfeeding up to six months 3. Colostrum feeding 4. Positioning and attachment 5. Management of breast complaints 6. Other (specify) |
| **Part IV: Maternal feeding practice of their neonates** | | | | |
| 27 | Did you give anything to drink and/or eat before breast feeding within 3 days for your neonate after delivery? | | | 1. Yes 2. No |
| If your answer to **Question 27** is ‘Yes’, answer the following three questions (Question 22-Question 24) | | | | |
| 28 | What did you give? (Multiple responses are possible) | | | 1. Plain water 2. Sugar water 3. Cow milk 4. Butter 5. Formula milk 6. Other (specify)----- |
| 29 | How did you give the feed? | | | 1. Spoon feeding 2. Finger feeding 3. Stick feeding 4. Other (specify)____ |
| 30 | Who did influence you to give the feeds? | | | 1. Traditional birth attendants 2. Grand parents 3. Own decision 4. Husband 5. Friends 6. Others (specify) |
| 31 | Do you have history of giving foods other than breast milk in the first 3 hours of birth to your prior child/children? | | | 1. Yes 2. No |
| Part V: Maternal information of feeding practice | | | | |
| 32 | Did you believe in the purported advantages of giving foods other than breast milk in the first 3 days of birth? | | | 1. Yes 2. No |
| 33 | If yes to **question 32**, what are these advantages of giving foods other than breast milk in the first 3 hours of birth? | | | 1. To clean the newborn’s bowel, throat or mouth 2. To quench neonatal thirst 3. To calm/sooth the baby 4. To improve neonatal health and growth 5. To nourish the neonate during any feeding problem 6. To nourish the neonate during maternal medical illness 7. To perpetuate cultural value 8. Other (specify)……. |
| 34 | Do you think giving foods other than breast milk in the first 3 hours of birth has risk to the neonate? | | | 1. Yes 2. No |
| 35 | If your answer to question 34 is yes, which of the following? | | | 1. Diarrhea 2. Poor growth 3. Infection 4. Vomiting 5. Others (specify) _____ |
| 36 | Did you colostrum feed your neonate? | | | 1. Yes 2. No |
| 37 | If your answer to question 36 is ‘No’, why? | | | 1. Causes abdominal discomfort and diarrhea 2. Inadequate breastmilk secretion 3. Maternal medical illness 4. Other (specify) ______ |
