# Supplementary material for: Prevalence and associated factors of prelacteal feeding among neonates admitted to neonatal intensive care units, North central Ethiopia, 2019
Source: BMC Public Health. 2020 Sep 25;20:1457. doi: 10.1186/s12889-020-09578-5 (PMC7519479; doi:10.1186/s12889-020-09578-5)
Supplement: Supplementary file 2 — Additional file 2: Supporting information 2. Survey questionnaire in Amharic (original) language. [file 12889_2020_9578_MOESM2_ESM.docx]

**የአማርኛ ቃለመጠይቅ**

**የሚስጥር ቁጥር: _______**

| **ክፍል አንድ፡ የማህበራዊ ጉዳዮችን በተመለከተ** | | |
| --- | --- | --- |
| **ተ/ቁ** | **ጥያቄ** | **መልስ** |
| 1 | የእናት መኖሪያ ቦታ | 1. ከተማ 2. ገጠር |
| 2 | የጋብቻ ሁኔታ | 1. ያላገባች 2. ያገባች 3. የፈታች 4. የሞተባት |
| 3 | ሃይማኖት | 1. ኦርቶዶክስ 2. ሙስሊም 3. ፕሮቴስታንት 4. አድቬንቲስት 5. ሌላ |
| 4 | የእናቲቱ እድሜ | ----------- (በዓመት) |
| 5 | የእናቲቱ የት/ት ደረጃ | 1. ማንበብና መፃፍ አትችልም 2. ማንበብና መፃፍ ትችላለች 3. ከ1ኛ-8ኛ ክፍል የተማረች 4. ከ9-12ኛ ክፍል የተማረች 5. ዲፕሎማ እና በላይ |
| 6 | የቤተሰብ አማካይ ወርሃዊ ገቢ | ____ ($USA) |
| 7 | ስንት ልጆች ወልደዋል? | 1. አንድ ብቻ 2. ከአንድ በላይ |
| 8 | ልጆችዎን በየስንት አመቱ ወለዱ? | 1. አንድ 2. ሁለት 3. ሶስት 4. አራት 5. ሌላ ካለ (ይጠቀስ) -------- |

**ክፍል ሁለት፡ የጨቅላ ህፃኑን መረጃ በተመለከተ**

| 9 | የህፃን አይነት | 1. ብቸኛ 2. መንትያ 3. ሶስትያ 4. አራትያ |
| --- | --- | --- |
| 10 | የህፃን ፆት | 1. ወንድ 2. ሴት |
| 11 | መረጃዉ በሚሰበሰብበት ጊዜ የጨቅላ ህፃንዎ ዕድሜ ስንት ነበር? | ---------------(በቀን) |
| 12 | ጨቅላ ህፃንዎን ከስንት ወር የእርግዝና ጊዜ በኋላ ወለዱ? | ------------- (በሳምንት) |
| 13 | የጨቅላ ህፃንዎ ክብደት ስንት ነዉ? | ---------------(በግራም) |
| 14 | ከዚህ በፊት ለጨቅላ ህፃንዎ ህክምና ወደዚህ ክፍል መጥተዉ ነበርን? | 1. አዎ 2. የለም |

| **ክፍል ሶስት፡** የእናቶች እና ህፃናት ጤና አገልግሎትን በተመለከተ | | |
| --- | --- | --- |
| 15 | ቅድመ ወሊድ ክትትል አድርገዋልን? | 1. አዎ 2. የለም |
| 16 | ለጥያቄ ቁጥር 15 መልስዎ አዎ ከሆነ፤ ስንት ጊዜ ቅድመ ወሊድ ክትትል አድርገዋል? | 1. አንድ 2. ሁለት 3. ሶስት 4. አራት 5. ከአራት በላይ |
| 17 | ቅድመ ወሊድ ክትትል ሲያደርጉ፣ ባለቤትዎ ኣብሮት ነበርን? | 1. አዎ 2. የለም |
| 18 | የት ነዉ የወለዱት? | 1. ጤና ተቋም 2. ቤት |
| 19 | በምን ነዉ የወለዱት? | 1. አምጠዉ 2. በቀዶ ጥገና |
| 20 | ማን ነዉ ያዋለደወት? | 1. ጤና ባለሙያ 2. የልምድ አዋላጅ 3. ሌላ-,------- |
| 21 | እርግዝናዎ ስንት ጨቅላ ልጅ አስገኘልዎ? | 1. አንድ 2. ሁለት 3. ሶስት 4. አራት |
| 22 | ጨቅላ ልጅዎን ጡት ማጥባት መቼ ጀመሩ? | 1. ጨቅላ ልጁ በተወለደ አንድ ሰአት ዉስጥ 2. ጨቅላ ልጁ ከተወለደ አንድ ሰአት በኋላ |
| 23 | የድህረ-ወሊደ ክትትል አድርገዋልን? | 1. አዎ 2. የለም |
| 24 | ስለ ጡት አጠባብ መርሆዎች የምክር አገልግሎት አግኝተዋልን? | 1. አዎ 2. የለም |
| 25 | ለጥያቄ ቁጥር 24 መልስዎ አዎ ከሆነ፣ መቼ ተመከሩ? | 1. በቅድመ ወሊድ ክትትል ጊዜ 2. በድህረ-ወሊደ ክትትል ጊዜ |
| 26 | ያገኙት የምክር አገልግሎት ምንን ይመለከታል? | 1. እስከ 6 ወር ጡት ብቻ 2. ስለ አንገር ጥቅም 3. ስለ ጡት አጠባብ ዘዴ 4. ስለ ጡት ህመም መፍትሄዎች 5. ሌላ ካለ (ይጠቀስ) -------- |

| **ክፍል አራት፡ እናት ለልጅዋ የምታደርገዉን የአመጋገብ ልምድ በተመለከተ** | | |
| --- | --- | --- |
| 27 | በወለዱ በ3 ሰአት ዉስጥ ከጡት በፊት ለልጅዎ የሚጠጣ/የሚበላ ነገር ሰጥተዋልን? | 1. አዎ  2. የለም |
| 28 | ለጥያቄ ቁጥር 27 መልስዎ አዎ ከሆነ ምን ሰጡት? | 1. የቧንቧ ዉሃ 2. የሱቅ ወተት 3. የስኳር ዉሃ 4. ቅቤ 5. የላም ወተት 6. ሌላ ካለ (ይጠቀስ) -------- |
| 29 | ለልጅዎ የሚጠጣ/የሚበላ ነገሩን በምን ቁስ ተጠቅመዉ ሊሰጡት ቻሉ ? | 1. በማንኪያ 2. በጣትዎ 3. በሹል እንጨት 4. ሌላ ካለ (ይጠቀስ) -------- |
| 30 | ይህንን አይነት ምግብ እንዲሰጡ ማን ነዉ የገፋፋዎት? | 1. የልምድ አዋላጅ 2. አያት 3. በራሴ ዉሳኔ 4. ባል 5. ጓደኛ 6. ሌላ ካለ (ይጠቀስ) -------- |
| 31 | ለከዚህ በፊት ጨቅላ ህፃንዎ ከጡት በፊት የሚጠጣ/የሚበላ ነገር ሰጥተዋልን? | 1. አዎ 2. የለም |
| **ክፍል አምስት፡ ከጡት ወተት በፊት ለጨቅላ ህፃናት የሚሰጡ ነገሮችን በሚመለከት እናቶች ያላቸዉ መረጃ** | | |
| 32 | ከጡት በፊት ለጨቅላ ህፃንዎ የሚጠጣ/የሚበላ ነገር መስጠት የሚያስገኛቸዉ ጥቅሞች አሉ ብለዉ ያምናሉ? | 1. አዎ 2. የለም |
| 33 | ለጥያቄ ቁጥር 32 መልሰዎ አዎ ከሆነ፤ ከሚከተሉት ዉስጥ ይምረጡ? (ከአንድ በላይ ይቻላል) | 1. የህፃኑን አንጀት ለማፅዳት 2. የህፃኑን የዉሃ ጥማት ለማርካት 3. ህጻኑን ዝም ለማስባል 4. ለህፃኑ እድገት 5. የህፃኑን የአመጋገብ ችግር ለማካካስ 6. እናት የጤና ችግር ሲገጥማት 7. ባህልን ለማስቀጠል 8. ሌላ ካለ (ይጠቀስ) -------- |
| 34 | ከጡት በፊት የሚሰጡ ነገሮች ለጨቅላ ህፃኑ ጎጂ ናቸዉ ብለዉ ያስባሉ? | 1. አዎ 2. የለም |
| 35 | ለጥያቄ ቁጥር 34 መልሰዎ አዎ ከሆነ ጉዳቱን ይግለፁ? | 1. ተቅማጥ 2. የእድገት መቀጨጭ 3. በሽታ 4. ትዉከት 5. ሌላ ካለ (ይጠቀስ) -------- |
| 36 | ጨቅላ ልጅዎን አንገር አጥብተዋልን? | 1. አዎ 2. የለም |
| 37 | ለጥያቄ ቁጥር 36 መልሰዎ የለም ከሆነ ለምን? | 1. በቂ ጡት ወተት አለመኖር 2. የእናት ህመም 3. ለጨቅላ ህፃኑ የሆድ ቁርጠት እና ተቅማጥ ስለሚያስከትል 4. ሌላ ካለ (ይጠቀስ) -------- |
